# Review of Randomized Controlled Trials of Massage in Preterm Infants

**DOI:** 10.3390/children4040021

**Published:** 2017-04-03

**Authors:** Anna-Kaisa Niemi

**Affiliations:** Department of Pediatrics, Division of Neonatal & Developmental Medicine, Stanford University, Palo Alto, CA 94304, USA; annakaisa.niemi@gmail.com; Tel.: +1-650-723-5711

**Keywords:** infant massage, preterm, premature, newborn, neonate, weight gain, hyperbilirubinemia, mind–body, randomized controlled trial, tactile-kinesthetic stimulation

## Abstract

Preterm birth affects about 10% of infants born in the United States. Massage therapy is being used in some neonatal intensive care units for its potential beneficial effects on preterm infants. This article reviews published randomized controlled trials on the effects of massage in preterm infants. Most studies evaluating the effect of massage in weight gain in premature infants suggest a positive effect on weight gain. Increase in vagal tone has been reported in infants who receive massage and has been suggested as a possible mechanism for improved weight gain. More studies are needed on the underlying mechanisms of the effects of massage therapy on weight gain in preterm infants. While some trials suggest improvements in developmental scores, decreased stress behavior, positive effects on immune system, improved pain tolerance and earlier discharge from the hospital, the number of such studies is small and further evidence is needed. Further studies, including randomized controlled trials, are needed on the effects of massage in preterm infants.

## 1. Introduction

Preterm birth (birth at <37 weeks of gestation) affects about 10% of infants born in the USA [1]. Premature infants often spend weeks or months in intensive care unit due to immaturity and need for intensive medical care, with hospital stay often prolonged by feeding immaturity and slow weight gain. Massage therapy is being used in some neonatal intensive care units for its possible beneficial effects with minimal side effects. This article reviews published randomized controlled trials on the effects of massage in preterm infants.

A PubMed search was performed for randomized controlled trials (RCTs) of massage in preterm infants. RCTs in English and indexed in PubMed by January 2017 were included. Studies assessing the effects of massage on caregivers only were excluded. A total of 34 randomized controlled trials on the effects of massage in preterm infants are reviewed [2,3,4,5,6,7,8,9,10,11,12,13,14,15,16,17,18,19,20,21,22,23,24,25,26,27,28,29,30,31,32,33,34,35].

## 2. Results

The outcome most commonly assessed on randomized controlled trials of massage in preterm infants (Table 1) was weight gain, either as primary or secondary outcome [2,5,8,9,10,12,15,16,17,18,19,21,26,28,29,30,31,33,34,35]. Other outcomes assessed in RCTs of massage in preterm infants include levels of transcutaneous bilirubin [3], sleep [4,26], calorie intake [5,17,19,30,33,34,35], vagal activity [5,19,24,26,29,30], gastric motility or number of stools [3,24,30], heart rate variability (HRV) [6,7,19,24,30], immunologic parameters [12], bone metabolism [11,32], changes in electroencephalogram (EEG) [13], behavior and/or neurodevelopment [14,15,23,26,28,29,31,34,35], pain [25], length of hospital stay [12,16,18] and levels of serum markers such as insulin-like growth factor I (IGF-1) [8,19], adiponectin [8], and serum triglycerides [9,27,31].

Most randomized controlled trials compared massage to standard care [2,3,4,6,7,8,9,11,12,13,14,16,17,18,19,20,21,22,23,24,25,26,27,28,29,30,31,32,33,34,35], while some compared oil massage to massage without oil [10], tactile massage to kinesthetic stimulation [5] and massage to light still touch [15]. While the type of massage used varied between studies, most studies assessed the effects of the tactile-kinesthetic stimulation (TKS) type of infant massage originally described by Field et al. in 1986 [36] or a modified version of TKS (Table 1). Modifications of TKS included shorter or longer duration of massage as well as elimination of kinesthetic range of movement exercise with only tactile stimulation provided. Other massage types evaluated include oil massage combined with either TKS or other standardized technique [2,9,10,27,28,31], Vimala massage [17] and acupressure and meridian massage [21].

In the following sections the outcomes of these studies as well as limitations are briefly reviewed. For number of study participants, gestational age, age at the time of study entry, and duration of massage intervention see Table 1.

### 2.1. Weight Gain

Weight gain, either as primary or secondary outcome is the most commonly assessed outcome in randomized controlled trials of massage in preterm infants [2,5,8,9,10,12,15,16,17,18,19,21,26,28,29,30,31,33,34,35]. Most studies have demonstrated a significantly greater daily or overall weight gain during the study period in massage group compared to control group [2,9,10,12,17,19,21,26,28,30,33,34,35] while some studies did not show a statistically significant difference in weight gain between groups [5,8,15,16,18,29,31]. Massaro et al. [16] evaluated the effects of massage or massage + kinesthetic stimulation on weight gain and length of hospital stay in a study of 59 infants who were randomized to massage only (n = 19), massage and kinesthetic stimulation (n = 20) or control group (n = 20). After controlling for covariates (birth weight, gestational age, caloric intake, etc.) the infants whose birth weight was >1000 g benefited from massage with a significantly higher average daily weight gain with more pronounced effects in infants who received massage + kinesthetic stimulation (*p* = 0.012) [16]. To study the possible contribution to weight gain of medium chain triglyceride (MCT) oil used in massage therapy (via absorption of oil through the skin) Saeidi et al. compared the effects of massage with MCT oil to massage without oil and controls (with no massage) and demonstrated a significanly higher weight gain during the 7-day study in the group that received massage with MCT oil compared to massage only (*p* = 0.002) and controls (*p* = 0.000) [2]. The mean weight gain during the study period in the MCT oil massage group was 105 ± 1.3 grams compared to a gain of 52 ± 0.1 grams in the massage only group and loss of 54 ± 1.3 grams in the control group [2]. 

### 2.2. Caloric Intake and Expenditure

Some studies have evaluated the effects of massage on caloric intake [5,8,17,19,30,33,34,35] and its possible connection to weight gain in preterm infants. Most studies did not show a difference in caloric intake between infants who received massage compared controls despite statistically significant higher weight gains in infants who received massage [8,17,19,30,33,34]. A study by Diego et al. showed increased calorie intake in infants who received kinesthetic stimulation compared to those who received tactile stimulation only, no difference in weight gain between the two groups [5]. Scafidi et al. evaluated factors that predict higher weight gain in infants who receive massage and a higher caloric intake was one of the factors that predicted weight gain [35]. One study evaluated energy expenditure in preterm infants, which was reported to be lower after a 5-day study period in infants who received massage compared to controls [22] suggesting that a decrease in energy expenditure may be in part responsible for the enhanced growth caused by massage therapy.

### 2.3. Vagal Tone

Increased vagal activity induced by massage has been suggested as one potential mechanism for higher weight gain in infants who receive massage therapy. Vagal activity in most studies is assessed by measuring heart rate variability (HRV) with increased high frequency (HF) variability indicating higher vagal tone [37]. All studies that evaluated vagal activity demonstrated an increase in vagal tone in infants who receive massage [5,6,7,19,24,26,29,30]. However, in two studies, the increased vagal effect was more pronounced in male infants [6] or was demonstrated only in males [7]. In a study by Diego et al. comparing the effects of tactile to kinesthetic stimulation (the two components of TKS massage [36]), an increase in vagal tone measured by HRV was only noted in in the tactile group in group-by-time analysis (*p* = 0.01), whereas a decreased vagal tone was noted in infants receiving kinesthetic range of motion excercises only [5]. 

### 2.4. Gastric Motility

Massage-induced increases in vagal activity may lead to increased gastric motility, which has also been suggested as a possible mechanism for increased weight gain in infants who receive massage therapy. Gastric motility or number of stools have been assessed in three studies [3,24,30]. In infants who received massage therapy, significantly increased gastric motility during and immediately after massage and decreased tachygastria has been reported [24,30]. This increase in gastric motility was significantly correlated with increased weight gain and vagal tone [24,30]. During a 4-day study which evaluated the effects of massage on transcutaneous bilirubin levels and stool frequency, a significantly increased stool frequency was reported in the massage group compared to controls during the entire study period (day 1: *p* = 0.001, day 2: *p* = 0.02, day 3: *p* = 0.01 and day 4: *p* = 0.04) [3]. 

### 2.5. Immunological Effects

Two randomized controlled studies have reported on the effects of massage in immunological parameters and incidence of infections in preterm infants [12,18]. Absolute number of natural killer (NK) cells, white blood cells, B and T cells, and T-cell subsets were not statistically different between the groups. Number of infections did not differ between the groups. While the mean absolute NK cell number was not statistically different between the massage and control group, the adjusted mean NK cell cytotoxicity was higher in the massage group (*p* = 0.05) [12]. Mendes et al. reported on significantly lower incidence of late-onset sepsis in massage group (*p* = 0.005) [18]. The incidence of late-onset sepsis was 38.3% (18/47 infants) in controls compared to 10.9% (5/46 infants) in massage group. No serum immunological markers were reported in this latter study. 

### 2.6. Bone Metabolism

Preterm infants have increased morbidity from osteopenia [38]. Physical activity stimulates bone formation. Two studies have evaluated the effects of massage therapy with physical activity on bone metabolism in preterm infants (Table 1) [11,32]. Physical activity was defined either as kinesthetic movement part of TKS massage [11] or a similar daily range of motion exercise, with gentle compression and extension/flexion to both upper and lower extremities [32]. Aly et al. reported an increase in serum type I collagen C-terminal propeptide (PICP, marker of bone formation) in the massage group (*p* < 0.01) compared to baseline, while PICP decreased in controls group (*p* < 0.01). This change differed significantly between the groups (*p* = 0.0001), suggesting increased bone formation in massage group. Furthermore, serum parathyroid hormone (PTH) level increased in the massage group while it decreased in the control group (*p* < 0.001). 

### 2.7. Behavior, Sleep and Neurodevelopment

Yates et al. studied the effects of TKS massage on sleep efficiency in preterm infants in a crossover study [4] and found no significant difference between groups for sleep efficiency (*p* = 0.13). More infants were reported to sleep on the non-massage day (*p* = 0.026) [4]. Several studies have assessed the effects of massage on behavior and/or neurodevelopment of preterm infants [14,15,23,26,28,29,31,34,35]. Most studies have reported on immediate behavioral effects of massage [15,23,26,28,29,31,34,35] and only one randomized controlled trial has reported on long term neurodevelopmental outcome [14]. Massage groups were reported to have significantly less stress-related behaviors (crying, fidgeting) than controls in three studies [23,26,34], no difference between the groups in two [28,31], and one study reported increased crying and fidgeting in infants who received massage [29]. Ho et al. reported on a higher gain in motor performance score (Test of Infant Motor Performance, TIMP) in very low birth weight (VLBW) infants who received massage and who had a below-average pre-study score compared to a control group who received light touch (*p* = 0.043) suggesting that massage may have positive effects in motor outcomes in a subgroup of VLBW infants who have low motor performance [15]. Procianoy et al. compared neurodevelopmental outcomes (Psychomotor Development Index, PDI and Mental Development Index, MDI) at 2 years of age between preterm infants who had received massage intervention during hospital stay and controls [14]. Infants who had been randomly assigned to massage therapy group or control group during their hospital stay both received skin-to-skin care. Growth and neurodevelopmental outcome were evaluated in both groups at 2 years corrected age. Growth at 2 years corrected age did not differ between the groups. The massage group had slightly but not significantly higher PDI score (*p* = 0.072) and significantly higher MDI scores (*p* = 0.035) than controls [14]. 

### 2.8. Length of Hospital Stay

Length of hospital stay (LOS) was reported to be similar in the massage group and controls in one study [12], and in one there was a significant difference between groups [16] but this difference disappeared after controlling for gestational age, gender, sepsis and birth weight. Two studies have reported shorter duration of hospitalization in infants who received massage compared to controls [17,18]. A study by Gonzalez et al. evaluating the effects of Vimala massage in preterm infants reported a shorter hospital stay in infants of massage group (15.36 ± 5.41 days) compared to controls (19.33 ± 7.92 days (*p* = 0.03) [17]. A study by Mendez et al. on VLBW infants reported that those who received massage had a 1.85 times higher (95% confidence interval (CI): 1.09 to 3.13; *p* = 0.023) probability of earlier hospital discharge than control group [18]. 

### 2.9. Serum Markers

Levels of serum markers of growth and metabolism such as IGF-1 [8,19], adiponectin [8], serum triglycerides [9,27,31] have been studied in small randomized controlled trials. Moyer-Mileur et al. studied IGF-1, leptin, and adiponectin levels in infants who received massage and controls and correlated them with skin fold thickness, weight gain and ponderal index (PI) [8]. Serum leptin levels correlated significantly with weight gain, PI, triceps skin-fold thickness and mid-thigh skin-fold thickness [8]. Serum adiponectin level correlated with PI. There was no difference in weight gain however in this study between massage and control groups. In a study by Field et al. the massage group had a greater increase in weight gain (*p* = 0.02), and a greater increase in both insulin (*p* = 0.001) and IGF-1 (*p* = 0.05) levels [19] and weight gain correlated with increased insulin (*p* = 0.05) and IGF-1 (*p* = 0.02) levels [19]. Three randomized controlled trials have evaluated the effects of oil massage on serum triglycerides [9,27,31]. The primary objective of Solanki et al. was to assess transcutaneous absorption of massage oils by assessing serum lipid profiles in infants assigned to a massage without oil, or with safflower oil (rich in essential fatty acids, EFAs) or coconut oil (rich in saturated fats and medium chain triglycerides, MCTs) [27]. No weight gain was measured in this study. Serum triglyceride levels were significantly higher after massage compared to baseline in all groups (coconut oil *p* < 0.001, safflower oil *p* < 0.001, controls *p* < 0.05) though the rise was significantly higher in oil groups compared to controls (*p* < 0.05). In infants randomized to massage with safflower oil, a significant rise was seen in the essential fatty acids linoleic and arachidonic acid (*p* < 0.001) after massage. In those who received a massage with coconut oil an increase in serum saturated fats was reported (*p* < 0.001). In the control group who received a massage without oil a small but significant increase in serum linoleic (*p* < 0.05) and total saturated fats was seen (*p* < 0.05) [27]. The other two studies that assessed serum triglyceride levels used sunflower oil in the massage group evaluated serum triglyceride levels as a secondary outcome and reported no significant difference between massage group and controls [9,31].

### 2.10. Bilirubin levels, markers of brain maturation, pain

Basiri-Moghadam et al. reported significantly lower transcutaneous bilirubin levels (*p* = 0.003) in the massage group compared to controls after four days of massage [3]. Guzzetta et al. reported differences in EEG spectral power (an index of brain maturation) in massaged infants compared to controls suggesting a process of brain maturation in those who received massage [13]. Jain et al. reported in a cross-over study higher scores in Neonatal Infant Pain Scale (primary outcome) (*p* < 0.001) and heart rate (*p* = 0.03) in infants who had a blood sampling via a heel stick without a preceding 2-min massage of the ipsilateral leg compared to infants who had a massage prior to heel stick [25]. Respiratory rate, oxygen saturation and serum cortisol levels did not differ [25].

## 3. Discussion

Preterm birth (birth at <37 weeks of gestational age) is the leading cause of neonatal morbidity and mortality in the United States [39,40] and affects 10% of infants born in the USA [1]. Slow weight gain and poor oral-motor ability to feed are common factors delaying discharge from hospital in infants born prematurely. Interventions that improve either weight gain or oral-motor function may lead to shorter hospital stay and cost savings. Improved weight gain is the most commonly and consistently reported effect of massage in preterm infants with significantly greater daily or overall weight gain found in infants who received massage compared to controls [2,9,10,12,17,19,21,26,28,30,33,34,35]. While one study reported increased gain in an infant motor performance score in VLBW infants who received massage suggesting positive effects on motor development [15], oral–motor coordination and ability to feed was not evaluated. To date there are no randomized controlled trials evaluating the effects of massage on oral–motor coordination or feeding ability offering an area of future studies.

A recent meta-analysis by Wang et al. concluded that massage improved daily weight gain by 5.32 g (95% CI 4.15, 6.49 g, *p* < 0.00001) and reduced length of hospital stay by 4.41 days (95% CI 2.81, 6.02 days, *p* < 0.00001) [41]. A Cochrane systematic review by Vickers et al. also concluded that massage improved daily weight gain by 5.1 g (95% CI 3.5, 6.7 g) and appeared to reduce length of hospital stay by 4.5 days (95% CI 2.4, 6.5 days) [42], consistent with meta-analysis by Wang et al. [41]. Furthermore, a review by Field et al also suggested that infant massage leads to 3–6 days shorter length of hospital stay and, consequently, significant cost-savings up to $10,000 per infant [43].

The mechanism by which massage improves weight gain in preterm infants is not well understood. One suggested mechanism is that massage leads to higher consumption of calories. However, studies have not shown a difference in caloric intake between infants who received massage compared controls [8,17,19,30,33,34]. Another possible mechanism is increased vagal tone which may promote food absorption and anabolism. Increase in vagal activity has indeed been consistently reported in infants who receive massage in all randomized controlled trials that evaluated vagal tone [5,6,7,19,24,26,29,30]. Levels of serum anabolic hormones insulin and IGF-1 have been reported to be higher in infants who had received massage and correlated with weight gain [19] suggesting a possible anabolic effect. Field et al. postulate in more detail potential mechanisms for improved weight gain in preterm infants who received massage, including increased vagal tone and IGF-1 levels [44].

Furthermore, increased weight gain in preterm infants who receive massage with oil may be due to transcutaneous absorption of oils and contribution to caloric intake. While Solanki et al. reported a significant increase in serum essential fatty acids and saturated fats in infants massaged with safflower and coconut oil, respectively, their study did not assess weight gain [27]. Thus, the possible effects of transcutaneous absorption of oil in weight gain could not be evaluated. Study by Saeidi et al. did demonstrate a significantly higher weight gain in infants who received massage with MCT oil compared to massage only or controls [2] suggesting that MCT oil, possibly via transcutaneous absorption, may partly contribute to weight gain.

Improved weight gain and decreased length of hospital stay are important measures on the effects of massage in preterm infants and may lead to significantly lower health care costs [43]. Yet little is known about the long-term effects of early massage on infant development. Only one small study (n = 73) has assessed the long-term effects of massage given during neonatal hospital stay and compared neurodevelopmental scores between infants who received massage therapy to controls [14] and reported on a slightly higher PDI score and significantly higher MDI scores in the massage group, suggesting massage may induce long-term effects in neural modeling and neurodevelopment. 

Overall, several studies as well as meta-analyses have suggested beneficial effects of massage in preterm infants with regards to improved weight gain and shortening the length of hospital, the latter of which has potential for significant cost-savings. Limitations and challenges of current randomized controlled trials on the effects of massage in preterm infants include small sample size in most studies, lack of studies in extremely premature infants, and lack of studies in infants with congenital anomalies or congenital heart disease who often have poor weight gain. The provider of the massage is also not consistent between studies. Massage has been variably provided by licensed massage therapists, trained nurses, or by a mother trained in massage technique. Although one study assessed the effects of massage in weight gain in infants massaged by either the mother or a professional gained and reported more weight gain in both groups compared to controls (*p* = 0.03) suggesting that different providers provide equally effective massage. There is limited information available on side effects of massage therapy with most of the studies not reporting side effects or quoting massage as “safe” or “relatively safe”. However, given lack of reported side effects it is likely safe to conclude that massage is “relatively safe” in the infant groups in which it has been studied. There is not enough evidence to recommend massage for all preterm infants but it may be beneficial in selected clinically-stable preterm infants with slow weight gain. 

## 4. Conclusions

In summary, randomized controlled trials on the effects of massage in preterm infants suggest improved weight gain and shortened length of hospital stay. Improved weight gain may be mediated through increase in vagal tone but more studies are needed on the underlying mechanisms of the effects of massage. Further randomized controlled studies are needed on the effects of massage on outcomes such as neurodevelopment, stress behavior, immune system, and pain tolerance.

## Figures and Tables

**Table 1:** Randomized controlled trials of massage in preterm infants.

Year	First Author	Number of Infants (Cases, Controls)	Gestational Age (Age at the Time of Study)	Massage Type	Intervention	Outcomes Measured	Results
2015	Basiri-Moghadam [3]	40 (20 massage, 20 controls)	34–36 weeks (<1 week)	N/A	20 min of massage twice daily for 4 days	Number of defecations Transcutaneous bilirubin level	Massage group had higher number of defecations (*p* = 0.002) and lower levels of transcutaneous bilirubin (*p* = 0.003)
2015	Saeadi [2]	121 (40 oil massage, 40 massage, 41 controls)	<37 weeks (<28 days)	N/A	5 min of massage 4 times a day for 7 days	Weight gain	The MCT oil-massage group gained more weight than massage (*p* = 0.002) and control groups (*p* = 0.000)
2014	Yates [4]	23 (cross-over study)	28–37 weeks (34–48 PMA)	TKS (modified, no kinesthetic component)	13 infants received a massage on day 1 and no massage on day 2 (10 infants received a massage on day 2)	Sleep efficiency Number of infants asleep at the end of massage	No significant difference in sleep efficiency (*p* = 0.13)
2014	Diego [5]	30 (15 tactile, 15 kinesthetic stimulation)	28–32 weeks; (15–60 days)	TKS (tactile or kinesthetic stimulation)	15 infants received tactile and 15 received kinesthetic stimulation 10 min a day for 5 days (no control group without massage)	Weight gain Calorie intake Vagal activity	No difference in weight gain. Increased calorie intake in kinesthetic group (*p* < 0.05). Increased vagal tone in tactile group (*p* = 0.01), decreased vagal tone in kinesthetic group.
2013	Fallah [10]	54 (17 oil massage, 17 massage alone) LBW infants	33–37 weeks (<10 days)	Moderate pressure	17 received moderate pressure massage alone, 17 received massage with sunflower oil 3 times a day for 14 days (no control group without massage)	Weight gain at 14 days, 1 month, 2 months	The oil massage group had a higher mean weight at ages 1 month (*p* = 0.04) and 2 months (*p* = 0.005) than the massage alone group.
2013	Smith [6]	21 (10 massage, 11 controls)	28–33 weeks (mean 31 weeks PMA)	Moderate pressure strokes with kinesthetic movement of extremities	20 min of massage twice daily for 4 weeks	Heart rate variability (HRV)	Significant group × time × sex interaction effect (*p* < 0.05) with male infants who received massage demonstrating higher HRV indicating increased vagal tone.
2013	Smith [7]	37 (17 massage, 20 control)	29–32 weeks	Soft-tissue compression strokes with following range of motion to arms and legs	20 min massage twice daily for 4 weeks	HRV	HRV improved in massage group but not in controls (*p* < 0.05). Male infants who received massage had a greater improvement in HRV than females (*p* < 0.05).
2013	Moyer-Mileur [8]	44 (22 massage, 22 controls)	29–32 weeks (32–33 weeks PMA)	Soft-tissue strokes with following range of motion to arms and legs	Twice-daily massage 6 days a week for 4 weeks	Energy and protein intake, body circumference, weight, length, ponderal index (PI), skinfold thickness (triceps, mid-thigh, subscapular), and IGF-1, leptin and adiponectin levels	Male infants in the massage group had smaller PI and skinfold thickness than control males (*p* < 0.05). Females in the massage group had larger subscapular skin fold increase than control females (*p* < 0.05). Adiponectin increased in control males (*p* < 0.01) and correlated to PI (r = 0.39, *p* < 0.01).
2013	Kumar [9]	48 (25 oil massage, 23 controls)	<35 weeks (<48 h)	20 strokes in: shoulders starting from neck with baby prone, upper back to the waist. Then limbs in supine position	10 min 4 times a day for 28 days	Weight, length, head circumference, serum triglyceride levels	At 7 days the massage group had less weight loss compared to controls (*p* = 0.003). At 28 days massage group had greater weight compared to controls (*p* < 0.05). No significant difference in serum triglycerides and other measured parameters.
2012	Haley [11]	40 (20 massage, 20 controls)	29–32 weeks, (mean 32 weeks PMA)	TKS	20 min twice daily, 6 days a week for 15 days	Tibial speed of sound (tSOS), urine markers of bone metabolism (pyridinium crosslinks and urinary osteocalcins (OC) U-MidOC and unOC)	Massage group had less decrease in tSOS than controls (*p* < 0.05). Urinary pyridinium crosslinks decreased in both massage and controls (*p* < 0.005). Massage group had greater increases in urinary osteocalcin (U-MidOC, *p* < 0.001 and unOC, *p* < 0.05) suggesting improved mineralization.
2012	Ang [12]	120 (58 massage, 62 controls)	Mean 30 weeks, range 25–33 (mean 32 weeks PMA, range 28–33)	TKS	5 days a week until hospital discharge for a maximum of 4 weeks	Immunologic parameters (absolute natural killer (NK) cells, T and B cells, T cell subsets, NK cytotoxicity), weight, number of infections, length of hospital stay	NK cytotoxicity was higher in the massage group, particularly in those who received ≥ 5 consecutive days of massage (*p* = 0.04). Infants in the massage group weighed more (*p* = 0.05) and had greater daily weight gain (*p* = 0.01) compared to controls. Other parameters, did not differ between the groups.
2011	Guzzetta [13]	20 (10 massage, 10 controls)	30–33 weeks (10 days)	TKS	10 min of massage 3 times a day for 12 days during a 2-week period	Changes in EEG spectral activity (before and after study period)	Significant difference in EEG spectral power.
2010	Procianoy [14]	73 (35 massage, 38 controls)	<32 weeks (48 h of life)	TKS (modified)	Massage and skin to skin care 15 min 4 times a day during hospital stay (skin to skin only)	Neurodevelopment (PDI, MDI) and growth at 2 years of corrected age	Massage group had higher MDI scores (*p* = 0.035), PDI scores and growth did not differ significantly.
2010	Ho [15]	24 (12 massage, 12 controls) VLBW infants	<34 weeks (34 weeks PMA)	TKS (modified)	15 min of massage daily, 5 days/week for 4 weeks (light still touch)	Test of Infant Motor Performance (TIMP) score gain, weight gain, post-conceptional age at discharge	No significant difference in TIMP score gain and weight gain when all subjects were analyzed. Those who had below-average pre-massage TIMP score and received massage had higher TIMP score gain (*p* = 0.043) and earlier discharge (*p* = 0.045) than controls.
2009	Massaro [16]	60 (20 massage with kinesthetic stimulation, 20 massage, 20 controls)	<32 weeks, mean 29 weeks for massage groups, 27 weeks for controls (30 weeks PMA)	TKS	15 min of massage ± kinesthetic stimulation (KS) twice daily from study enrollment until discharge	Weight gain Length of stay (LOS)	Average daily weight gain and LOS did not differ between the groups except for infants with BW > 1000 g, in whom average daily weight gain was higher in massage + KS group compared to control.
2009	Gonzalez [17]	60 (30 massage, 30 controls)	<35 weeks, mean 31 weeks (30–35 weeks PMA, mean 33)	Vimala massage	15–20 min twice daily massage for 10 days	Weight, head circumference, caloric intake, nutritional method, hospital stay	Massage group had higher weight gain (*p* < 0.001) and shorter hospital stay (*p* = 0.03) than controls.
2008	Field [19]	42 (N/A)	Mean 29–30 weeks (mean 34.8 weeks PMA)	TKS	15 min per day for 5 days	Weight gain, caloric consumption, vagal activity (high frequency component of HRV), serum insulin and IGF-1	Vagal activity increased during massage (*p* < 0.001). Massage group had greater increase in weight gain (*p* = 0.02), insulin (*p* = 0.001) and IGF-1 (*p* = 0.05). No difference in caloric consumption was noted between groups.
2008	Mendes [18]	104 (52 massage, 52 controls) VLBW infants	<32 weeks, mean 29 weeks (48 h)	TKS (modified)	15 min 4 times a day until discharge	Length of hospital stay (primary). Other outcomes: weight, length, head circumference, growth rate, ponderal index, age of partial and total enteral feeding, age of partial and total oral feeding, incidence of late onset sepsis, necrotizing enterocolitis (NEC), bronchopulmonary dysplasia (BPD)	Massage group had a higher probability of earlier hospital discharge (*p* = 0.023). Incidence of late-onset sepsis was lower in the massage group (*p* = 0.005).
2008	Chen [21]	40 (20 massage, 20 controls)	<34 weeks (>7 days)	Acupressure and meridian massage	15 min 3 times a day for 10 days	Weight gain	During 14-day study period the massage group gained more weight (*p* = 0.038) There was no significant difference in weight gain in the first week (*p* = 0.384). In the second week, the massage group had had higher weight gain than controls (*p* = 0.035).
2008	Diego [20]	72 (abstract) or 48 (methods)(N/A)	Mean 29 weeks (N/A)	TKS	15 min of massage once	Temperature (15 min before, during massage and 15 min after)	Massage group had a greater increase in temperature
2007	Lahat [22]	10 (cross over)	Mean 32 weeks (29–34 weeks PMA, mean 3 weeks)	TKS (modified)	15 min 3 times a day for 5 days (5 infants received a massage for 5 days and no massage for 5 days, opposite sequence for 5 infants)	Energy expenditure by indirect calorimetry	Energy expenditure was lower in infants after the 5 days massage therapy than after the period with no massage (*p* = 0.05).
2007	Diego [24]	70 (36 massage, 34 controls)	Mean 29 weeks (mean 30 days)	TKS	15 min 3 times a day for 5 days	Vagal activity (HRV). Gastric motility (by EGG) measurements performed on days 1 and 5 of study 15 min before, during and 15 min after massage	Group-by-time analysis revealed significant increase in vagal activity that peaked during massage (*p* < 0.001); a significant increase in gastric motility that peaked 15 min after massage (*p* < 0.01) in the massage group. No changes in basal vagal activity or gastric motility were noted.
2007	Hernandez-Reif [23]	32 (16 massage, 16 controls)	28–32 weeks (15–60 days)	TKS	15 min 3 times a day for 5 days	Stress behavior (crying, grimacing, yawning, jerky arm or leg movement, sneezing, startles, finger flaring). Activity (movement of the limbs, torso or gross body movement of any kind)	Group-by-time analysis showed reduction in duration of stress behaviors in massage group (*p* < 0.05) and reduction in overall movement for the massage group (*p* < 0.05). No change in controls in these parameters were observed.
2006	Field [26]	68 (N/A)	28–32 weeks, mean 30 weeks (15–60 days, mean 23)	TKS	15 min 3 times a day for 5 days of moderate vs. light pressure massage (no control group without massage)	Behavior state, stress behaviors, heart rate, weight gain	Moderate pressure massage group had a higher increase in weight gain (*p* < 0.02), less decrease in deep sleep (*p* < 0.05), less increase in active sleep (*p* < 0.02), less increase in fussing (*p* < 0.01), less increase in crying (*p* < 0.02), less increase in gross movement (*p* < 0.05), less increase in stress behavior (*p* < 0.01), greater decrease in heart rate (*p* < 0.01), and greater increase in vagal tone (*p* < 0.05).
2006	Jain [25]	23 (crossover)	<37 weeks (1–7 days)	Slow massage of outer aspect of the leg	2-min massage of the ipsilateral leg prior to heel stick (n = 13) and no massage prior to next heel stick 2–7 days later (10 infants had reverse order)	Neonatal Infant Pain Scale NIPS (primary outcome) Heart rate, respiratory rate, oxygen saturation	NIPS (*p* < 0.001) and heart rate (*p* = 0.03) were higher after a heel stick with no massage.
2005	Arora [31]	69 (23 oil massage, 23 massage, 23 controls) VLBW infants	<37 weeks	Standardized technique for the study as described in article	10 min 4 times a day	Weight gain at 28 days after enrolment (primary), length, head circumference triceps skin fold thickness, neurobehavior (NBAS), serum triglycerides.	No significant differences in any of the measured parameters. Trend for increased weight gain/day in massage group (*p* = 0.07).
2005	Sankaranara-yanan [28]	112 (38 coconut oil, 37 mineral oil, 37 controls)	Mean 34.8 weeks (day 2 of life)	TKS	Four times a day for 31 days.	Weight gain velocity over the first 31 days of life (primary), length gain velocity, head growth, neuro-behavioral outcome, incidence of adverse events	Coconut oil massage group had a greater weight gain velocity compared to mineral oil (*p* < 0.05) and control groups (*p* < 0.05). Infants receiving coconut oil massage also showed a greater length gain velocity compared to controls (*p* < 0.05). No significant difference in other measures.
2005	Diego [30]	48 (16 massage, 16 light pressure sham massage, 16 controls)	22–37 weeks, mean ~30 (9–76 days, mean 29–34, no significant N difference between groups)	TKS	15 min 3 times a day for 5 days	Weight gain, vagal activity (HRV), gastric motility (EGG), days to discharge, caloric intake	Massage group had greater weight gain (*p* < 0.01), increased vagal tone (*p* < 0.05) and increased gastric motility (*p* < 0.01) during and after treatment. Weight gain was significantly related to gastric motility (*p* < 0.01) and vagal tone (*p* < 0.01). No differences in other parameters.
2005	Lee [29]	26 (13 massage, 13 controls)	< 36 weeks (second day after starting enteral feeds)	TKS	15 min massage twice daily for 10 days	Weight, vagal tone, heart rate, oxygen saturation, and behavioral responses (behavioral states, motor activity, behavioral distress)	Massage group had significantly higher vagal tone after massage (*p* = 0.05) no change in controls. Massage group had higher scores for awake state (*p* = 0.000), fidgeting or crying (*p* = 0.04), and motor activity (*p* = 0.04) than controls.
2005	Solanki [27]	120 (40 coconut oil, 40 safflower oil, 40 no oil)	Three groups: GA < 34 weeks, GA 34–37 weeks, GA > 37 weeks	Not specified	5 mL of oil massaged for 10 min 4 times a day for 5 days	Serum triglycerides, serum fatty acid profile (linoleic, arachidonic, alpha linolenic acid, docosahexaenoic acids and saturated fats)	Serum triglyceride values were significantly higher after massage in all groups compared to baseline (coconut oil *p* < 0.001, safflower oil *p* < 0.001, controls *p* < 0.05). There was a significant rise in linoleic and arachidonic acid in the safflower oil group (*p* < 0.001) and saturated fats in coconut oil group (*p* < 0.001).
2004	Aly [32]	30 (15 massage, 15 controls)	28–35 weeks (< 2 weeks)	TKS	Daily massage until infant reached 1.8 kg of weight.	Serum type I collagen C-terminal propeptide (PICP) and urinary pyridinoline crosslinks of collagen (Pyd)	Serum PICP increased in massage group (*p* < 0.01) and decreased in control group (*p* < 0.01) compared to baseline, and this change differed significantly between massage group and controls (*p* = 0.0001). Urinary Pyd increased in both groups compared to baseline (*p* < 0.01 for both) but did not differ between the groups.
2002	Ferber [33]	57 (21 massage by mother, 17 massage by professional, 19 controls)	26–34 weeks, mean 31 weeks (mean 12–17 days)	TKS (modified)	15 min massage 3 times a day for 10 days	Weight gain Calorie intake	Infants massaged by either their mother or professional gained more weight than controls (*p* = 0.03). No significant difference in caloric intake.
1993	Wheeden [34]	30 (15 massage, 15 controls) All cocaine exposed infants	<37 weeks, mean 30 weeks (mean 16 days in controls, 20 days in massage group, NS)	TKS	15 min massage 3 times a day for 10 days	Weight gain, calorie intake, stress behavior, behavior (NBAS)	Massage group had greater weight gain per day (*p* = 0.009) than controls. There was no difference in feed type or caloric intake. Massage group had fewer postnatal complications (*p* = 0.005) and stress behaviors (*p* = 0.05) and more mature motor behaviors at the end of the 10-day study period (*p* = 0.02) than controls.
1993	Scafidi [35]	93 (50 massage, 43 controls)	26–36 weeks, mean 30 weeks (mean 15 days)	TKS	15 min massage 3 times a day for 10 days	Weight gain, calorie intake, stress behavior, behavior (NBAS)	Massage group had greater average daily weight gain (*p* < 0.01) than controls. Factors that predicted higher weight gain were history of obstetric complications, higher caloric intake and longer stay in intermediate nursery.

Controls received standard care unless otherwise noted. Statistically significant differences are noted in “Results” column, for other outcomes measured the difference was not statistically significant. BW = birth weight, EEG = electroencephalogram, EGG = electrogastrogram, GA = gestational age, HRV = heart rate variability, IGF-1 = insulin-like growth factor I, LBW = low birth weight, MCT = medium chain triglycerides, MDI = mental development index, NBAS = Brazelton’s neonatal behavior assessment scale, NS = , PDI = Psychomotor Development Index, PMA = post-menstrual age, TKS = tactile/kinesthetic stimulation, a massage technique originally described by Field in 1986 [36], U-MidOC = urine osteocalcin midfragments, unOC = undercarboxylated form of osteocalcin, VLBW = very low birth weight.
